# Psychometric Properties of the Chinese Version of the Neuroticism Subscale of the NEO-PI

**DOI:** 10.3389/fpsyg.2018.01454

**Published:** 2018-08-17

**Authors:** Chang Xi, Mingtian Zhong, Xiaoxia Lei, Ying Liu, Yu Ling, Xiongzhao Zhu, Shuqiao Yao, Jinyao Yi

**Affiliations:** ^1^Medical Psychological Center, The Second Xiangya Hospital, Central South University, Changsha, China; ^2^Center for Studies of Psychological Application, School of Psychology, South China Normal University, Guangzhou, China; ^3^Education Institute, Hunan Agricultural University, Changsha, China; ^4^Medical Psychological Institute of Central South University, Changsha, China

**Keywords:** neuroticism, big five, measurement equivalence, factor structure, reliability, validity

## Abstract

Neuroticism is an important concept in psychology, self-report measures of neuroticism are important for both research and clinical practice. The neuroticism subscale of the Neuroticism-Extraversion-Openness Personality Inventory (NEO-PI) is a brief measure of neuroticism, and it was widely used in the world. This study was aimed to examine the psychometric properties of the Chinese version of the neuroticism subscale of the NEO-PI. A total of 5,494 undergraduates from three universities and 551 clinical patients with mental disorders from a psychological clinic had completed the Chinese version of the neuroticism subscale of the NEO-PI. Confirmatory factor analysis was performed to examine how well the three hypothetical models fit the data and the measurement equivalence of neuroticism subscale across gender. The internal consistency and test-retest reliability were also evaluated. Both the six-facet model and the bi-factor model (six-facet model with one general factor) achieved satisfactory fit, while the six-facet model had best fit (Undergraduate sample: TLI = 0.919, CFI = 0.933, RMSEA = 0.044, SRMR = 0.033; Clinical sample: TLI = 0.921, CFI = 0.935, RMSEA = 0.047, SRMR = 0.041), and it had measurement equivalence across gender. The neuroticism subscale also showed acceptable internal consistency and good stability. Within the undergraduate sample, there were statistically significant gender differences in neuroticism total scores and scores of six facets, while there were no significant gender differences in the neuroticism scores in the clinical sample. Both in the undergraduate sample and the clinical sample, anxiety facet, depression facet and vulnerability facet of the neuroticism subscale significantly predicted the depression level, while anxiety facet, angry-hostility facet and vulnerability facet significantly predicted the anxiety level. In conclusion, the Chinese version of the neuroticism subscale is a reliable and valid measurement of neuroticism in both undergraduate and clinical population.

## Introduction

While the term neuroticism dates back to Freudian theory, the modern concept of neuroticism was introduced by Eysenck. Eysenck recognized neuroticism as a trait of emotionality, specifically the tendency to arouse quickly when stimulated and to inhibit emotions slowly (Eysenck and Michael, 1985). Neuroticism is operationally defined by the factors of items referring to irritability, anger, sadness, anxiety, worry, hostility, and self-consciousness (Costa and McCrae, 1992b).

Researches on neuroticism are important to public health due to the robust correlation between neuroticism and a wide variety of both mental and physical health problems (Malouff et al., 2005, 2006). Neurotic individuals have a limited tolerance for aversive stimuli and tend to experience negative emotion, such as anger, anxiety and depression (McCrae and Costa, 1997, 2008). Individuals with higher level of neuroticism not only tend to develop mood disorders, but also exhibit other disorders, such as substance use disorder and eating disorders (Widiger, 2001). Meanwhile, neurotic individuals are at higher risk of engaging in potentially destructive behaviors that may have a negative impact on health and lead to a decrease in life expectancy, such as smoking and excessive drinking (Daniel et al., 2009).

Self-reported questionnaires are the most commonly used tools for neuroticism assessment. The Eysenck Personality Questionnaire (Eysenck et al., 1985), the Neuroticism-Extraversion-Openness Personality Inventory (NEO-PI; John and Kentle, 1991), and the Dutch Personality Questionnaire (Kerkhof, 2003) have proven adequate psychometric properties in assessing neuroticism. The most widely used instrument to date is the 48-item neuroticism subscale of the NEO-PI, which assesses individual differences in a predisposition to experience negative emotional states associated with symptoms of depression, anxiety and high arousal (Pervin, 1999; Matthews, 2000). The NEO-PI is a 240-item self-reported questionnaire based on the five-factor model (the Big Five) of personality: Extraversion, Neuroticism, Conscientiousness, Agreeableness, and Openness/ Intellect. Each factor of NEO-PI includes six facets. Over the past two decades, the Big Five factors have become the most prominent model for describing the structure of personality traits (Egger et al., 2003; Malouff et al., 2006; Ortet et al., 2012). Among the Big Five factors, neuroticism has been the most studied factor by researchers around the world and the neuroticism subscale of the NEO-PI has been the most widely used instrument for the measurement of neuroticism. The neuroticism subscale of the NEO-PI measures six facets of neuroticism: anxiety, angry-hostility, depression, self-consciousness, impulsiveness and vulnerability (Costa and McCrae, 1992b). The psychometric properties of the NEO-PI scale have been proven validity across cultures and ages, each facet scale includes eight items to ensure that the scales are comparable in many respects (Terracciano, 2011). Considering the wide use of the neuroticism subscale of the NEO-PI, studies focusing on the psychometric properties of the neuroticism subscale are warranted.

Prior researches have found that women scored higher than men on almost all facets of neuroticism and on total level of neuroticism (Grossman and Wood, 1993; Lynn and Martin, 1997; Costa et al., 2001; Terracciano, 2003). One study including data from 26 countries concluded that women scored higher than men on neuroticism as measured by the neuroticism subscale of the NEO-PI, as well as on most facets of neuroticism (Costa et al., 2001). A study in Italy also found that women scored significantly higher than men on total neuroticism and on each of its facets (Terracciano, 2003). Since neuroticism increases the risk of a wide range of mental disorders, gender difference in neuroticism may lead to the gender difference seen in major psychopathologies. For example, women diagnosed with many mental disorders (e.g., phobias, major depression, dysthymic disorder, generalized anxiety disorder, borderline personality disorder) were associated with neuroticism at a higher rate than men (Grossman and Wood, 1993). Although there were many previous researches supporting the difference in neuroticism between men and women(Foot and Koszycki, 2010; Banzhaf et al., 2012; Konishi et al., 2014), only when the latent structure are equivalent across gender, the observed gender difference in neuroticism can truly predict the true difference between males and females.

Measurement invariance is “the mathematical equality of corresponding measurement parameters for a given factorially defined construct across groups” (Little, 1997, p. 55). Generally, measurement invariance is used to estimate parameters that reflect difference in the latent construct, and examine whether an instrument's items operate in the same way in different groups (Meredith, 1993). Simply put, it is used to examine whether an instrument's items measure the same parameters in different groups. If there is poor measurement invariance in the neuroticism scale across gender, the observed difference between males and females may be confounded by the fact that different constructs are being measured.

Therefore, the purpose of this study was to examine the psychometric properties of the 48-item neuroticism subscale of the NEO-PI in Chinese population. One undergraduate sample and one clinical sample were used in this study. Specifically, the current study would examine the measurement invariance across gender in the undergraduate sample.

## Methods

### Participants

From March 2015 to October 2015, 5,612 undergraduates from three universities in Changsha were invited to participate in the study. A total of 5,494 (99.7%) students [2,878 (52.4%) men, 2,616 (47.6%) women; aged 19–30 (Mean = 25.0; standard deviation (*SD*) = 1.02)] provided complete data anonymously. To estimate test–retest reliability, a subgroup of 865 students who had been participating in a psychology course completed the neuroticism subscale of the NEO-PI twice with a 2-month interval.

In addition to the subjects described above, from March 2015 to March 2017, 568 outpatients, who had been referred for assessment and treatment in the psychological clinic of Second Xiangya Hospital, were also asked to participate in the study. Those patients who could not understand the question well were excluded, such as patients with intellectual disability. A total of 551 (97%) outpatients provided complete data, consisting of 281 men (51%) and 270 women (49%), ranging in age from 16 to 77 (Mean = 31.41; *SD* = 18.52). The diagnoses of the clinical sample were schizophrenia, depressive disorder, anxiety disorder, obsessive-compulsive disorder, trauma and stress-related disorder, somatic symptom and related disorder, personality disorder and other mental disorders. There was a significant age difference between the undergraduate sample and the clinical sample, the clinical sample was significantly older than the undergraduate sample (*t* = 5.772, *p* < 0.001), but no significant gender difference was found between the two samples (χ^2^ = 0.386, *df* = 1, *p* = 0.535).

The study was approved by the Ethics committee of Second Xiangya Hospital, Central South University. All participants provided written informed consent at the time of enrollment.

### Instruments

#### The neuroticism subscale of the NEO-PI

The NEO-PI is the most comprehensive self-report questionnaire that measures the five-factor model of personality, in which neuroticism is included (Costa and McCrae, 1997; Xu and Potenza, 2012). The NEO-PI consists of 240 items and has been extensively validated (John and Kentle, 1991; Luchetti et al., 2018). It measures five major factors and 30 facets. The internal consistency of the NEO-PI was high (0.86 to 0.92), and the internal consistency of its facets ranged from 0.58 to 0.81 (Costa and McCrae, 1992a). The neuroticism subscale of the NEO-PI includes 48 items, each is rated on a 5-point Likert scale (0–4), with total score ranging from 0 to 192. Higher scores are indicative of higher level of neuroticism. The neuroticism subscale includes six facets: anxiety, angry-hostility, depression, self-consciousness, impulsiveness, and vulnerability. Each facet is measured by eight items, and the score of each facet ranges from 0 to 32 (Dai and Yao, 2004). The neuroticism subscale of the NEO-PI has previously been proven to be valid in the assessment of neuroticism among Chinese people (Dai et al., 1999).

#### The center for epidemiological studies depression scale

The 20-item Center for Epidemiological Studies Depression Scale (CES-D; Radloff, 1977) assesses various current depressive symptoms in general population. Each item is rated on a 4-point scale. Total score ranges from 0 to 80. Higher scores are indicative of a greater presence of depressive symptoms. The Chinese version of the CES-D has acceptable reliability (Cronbach's α = 0.88) and validity in Chinese population (Wang et al., 2013).

#### The self-rating anxiety scale

The Zung Self-Rating Anxiety Scale (SAS) was designed to quantify an indivdual's level of anxiety (Zung, 1965). Each question on the SAS is scored on a Likert-type scale of 1–4 (1 = “ rarely,” 2 = “some of the time,” 3 = “often,” and 4 = “most of the time”). The total score ranges from 20 to 80. Higher scores are indicative of more symptoms of anxiety. The SAS has demonstrated acceptable reliability (Cronbach's α = 0.78) and validity in the assessment of anxiety in Chinese-speaking samples (Luo et al., 2006; Wang and Tang, 2011).

## Statistical analyses

To evaluate the construct validity of the neuroticism subscale, we performed robust maximum-likelihood(MLR) confirmatory factor analysis (CFA) using M-plus 7.11 software. The CFA was used to determine how well the factor models fit the data, and to examine the measurement equivalence of neuroticism subscale across gender. We examined three hypothetical models: the original single-factor model (one general factor), the six-facet model (six facets: anxiety, angry-hostility, depression, self-consciousness, impulsiveness, and vulnerability), and the bi-factor model (six-facet model with one general factor, each item loading on both one facet and the general factor) (Reise et al., 2010). Each model included 24 parcels, which were created by randomly selecting two items within a facet for each parcel (Kishton and Widamen, 1994; Floyd and Widamen, 1995), so each facet included four parcels, see Supplementary Material. Several model fit indices were used to evaluate the model fit: tucker-lewis index (TLI), comparative fit index (CFI), and root-mean-square error of approximation (RMSEA) (Chou et al., 1991; Hu and Bentler, 1998). The criteria used to evaluate model fit were: TLI ≥ 0.90, CFI ≥ 0.90, and RMSEA ≤ 0.08 (Browne and Cudeck, 1993; Hu and Bentler, 1999). To further compare the fit of the competing models, the χ^2^ test is also reported.

To assess the measurement invariance of neuroticism subscale across gender in the undergraduate sample, the multi-group confirmatory factor analysis (MGCFA) was used. The test of measurement invariance was divided into four levels from low to high: configural invariance, weak factorial invariance, strong factorial invariance, and strict invariance (Joreskog, 1971). The four levels have a hierarchical relationship so that data analyses were carried out step by step: (1) configural invariance (model 1), a baseline model for each group was construed such that for both men and women the following criteria were met: TLI ≥ 0.90, CFI ≥ 0.90, and RMSEA ≤ 0.08, (2) weak factorial invariance (model 2), factor loadings were equal across groups, (3) strong factorial invariance (model 3), the factor loadings and intercepts of variables were equal across groups and (4) strict invariance (model 4), the factor loadings, intercepts of variables and error variances were equal across groups, while strict invariance is not necessary for most researches(Widaman and Reise, 1997). Measurement invariance is considered established when two of following are satisfied: the χ^2^ difference test resulted in a *p*-value > 0.05, the change of CFI < 0.01, the change of TLI < 0.01 and the change of RMSEA < 0.015(Cheung and Rensvold, 2002; Chen, 2007; Ferro and Boyle, 2013).

Cronbach's alphas (α) and mean inter-item correlations (M_IC_) for the full neuroticism scale and for each of the facets were calculated to evaluate internal reliability. Generally speaking, a minimum standard of 0.70 is set for Cronbach's α coefficients, but an α of 0.60 is also considered acceptable (DeVellis, 1991). An optimal range of 0.10–0.40 was set for the M_IC_ (Briggs and Venue, 1986; Nunnally, 1994). The Pearson correlation coefficient (*r*) was used to evaluate test–retest reliability and was set with a minimum standard of 0.70 (Anastasi and Urbina, 1997). The relationships between the total scale and the six facets were also examined by Pearson's *r*. Independent-samples *t*-test was used to compare the differences in scores of neuroticism subscale between the undergraduate sample and the clinical sample, and the gender differences in scores of neuroticism subscale.

To examine whether depression and anxiety were predicted by demographic variables and neuroticism, we performed multiple linear regression both in the undergraduate sample and the clinical sample, with the CES-D total score and SAS total score as dependent variable, respectively.

## Results

### Descriptive statistics

The total scores of neuroticism subscale of the NEO-PI ranged from 11 to 155 (Mean = 72.12; *SD* = 20.99) in the undergraduate sample. While in the clinical sample, the total scores of neuroticism subscale ranged from 19 to 175 (Mean = 106.84; *SD* = 28.36).

### The goodness of fit indices for neuroticism subscale

Both in the undergraduate sample and the clinical sample, the fit indices of the six-facet model and the bi-factor model (six-facet model with one general factor) all reached acceptable standards, but the single-factor model didn't fit quite well (Table 1). However, the six-facet model had significant improvement over the bi-factor model in the clinical sample, χ^2^ (diff) = 24.79, *p* < 0.001, but not in the undergraduate sample χ^2^ (diff) = 2.78, *p* > 0.05. The standardized factor loading of the six-facet model were presented in Table 2.

**Table 1:** The model fit indices of the six-facet model, the single-factor model and the six-facet bi-factor model in the two groups.

**Models**	**Undergraduate sample (*N* = 5,494)**			**Clinical sample(*****N*** = **551)**		
	**Six-facet**	**Single-factor**	**Bi-factor**	**Six-facet**	**Single-factor**	**Bi-factor**
S-Bχ^2^	3080.5594	4055.9917	3077.7788	603.0128	804.0263	627.8021
Df	237	252	228	237	252	228
TLI	0.919	0.899	0.915	0.921	0.887	0.911
CFI	0.933	0.910	0.932	0.935	0.900	0.929
RMSEA	0.044	0.049	0.045	0.047	0.056	0.050
SRMR	0.033	0.038	0.034	0.041	0.046	0.041
BIC	265683.798	266528.180	265756.601	31802.734	31909.091	31884.332

*S-Bχ2, Chi-Square Test of Model Fit; Df, degrees of freedom; GFI, The Goodness-of-fit Index; TLI, Tucker-Lewis Index; CFI, Comparative Fit Index; RMSEA, Root-Mean-Square Error of Approximation; SRMR, Standardized Root Mean Square Residual; BIC, Bayesian information criterion; Six-facet, The six-facet model; Single-factor, The single-factor model; Bi-factor, The bi-factor model (six-facet model with one general factor, each item loading on both one facet and the general factor)*.

**Table 2:** The factor loading of each parcel in the six-facet model.

	**Undergraduate sample**	**Clinical sample**		**Undergraduate sample**	**Clinical sample**
P1	0.522	0.596	P13	0.476	0.447
P2	0.690	0.733	P14	0.593	0.478
P3	0.533	0.650	P15	0.526	0.579
P4	0.588	0.670	P16	0.413	0.464
P5	0.560	0.576	P17	0.448	0.507
P6	0.444	0.407	P18	0.426	0.410
P7	0.446	0.536	P19	0.563	0.512
P8	0.707	0.750	P20	0.679	0.777
P9	0.536	0.756	P21	0.659	0.705
P10	0.665	0.761	P22	0.703	0.765
P11	0.575	0.682	P23	0.692	0.706
P12	0.700	0.778	P24	0.663	0.695

*P1~P4 belong to anxiety facet; P5~P8 belong to angry-hostility facet; P9~P12 belong to depression facet; P13~P16 self-consciousness facet; P17~P20 belong to impulsiveness facet; P21~P24 belong to vulnerability facet*.

### Measurement invariance across gender for the undergraduate sample

As the six-facet model fitted the data best, we choose the six-facet model to estimate the measurement invariance across gender. As shown in Table 3, the baseline models were considered optimal in representing the data both for the male (TLI = 0.917; CFI = 0.931; RMSEA = 0.041) and the female undergraduates (TLI = 0.915; CFI = 0.930; RMSEA = 0.047), providing evidence for configural invariance. Furthermore, the changes in TLI and RMSEA (ΔTLI < 0.010, ΔRMSEA < 0.015) supported weak equivalence and strong equivalence of the neuroticism subscale. In other words, the neuroticism subscale demonstrated good measurement equivalence across gender.

**Table 3:** Measurement invariance of the neuroticism subscale across gender in the undergraduate sample.

**Model**	**S-Bχ^2^**	**Df**	**TLI**	**CFI**	**RMSEA**	**SRMR**	**ΔTLI**	**ΔCFI**	**ΔRMSEA**
Model_male_	1548.9178	95	0.917	0.931	0.041	0.033			
Model_female_	1781.5871	95	0.915	0.930	0.047	0.037			
Model1	3426.8285	184	0.914	0.928	0.044	0.036			
Model2	3429.6562	169	0.917	0.928	0.043	0.036	0.003	0.000	−0.001
Model3	3818.7461	154	0.909	0.918	0.045	0.040	−0.008	−0.010	0.002
Model4	4353.0741	130	0.899	0.905	0.048	0.043	−0.010	−0.013	0.003

*Model 1, configural equivalence; Model 2, metric equivalence; Model 3, strong equivalence; Model 4, strict equivalence*.

### Reliability

In the undergraduate sample, the Cronbach's α coefficient was 0.91 for the neuroticism subscale, and ranged from 0.58 (self-consciousness) to 0.77 (vulnerability) for the six facets. In the clinical sample, the Cronbach's α coefficient was 0.93 for the neuroticism subscale, and ranged from 0.54 (self-consciousness) to 0.83 (depression) for its six facets (Table 4).

**Table 4:** The Cronbach's α, mean inter-item correlation and test-retest reliability for the neuroticism subscale and its six facets.

	**Undergraduate sample**			**Clinical sample**	
	**Cronbach's α (*N* = 5,494)**	**M_IC_ (*N* = 5,494)**	**Test–retest coefficient (*N* = 865)**	**Cronbach's α (*N* = 551)**	**M_IC_ (*N* = 551)**
Neuroticism	0.91	0.18	0.71	0.93	0.21
Anxiety	0.67	0.21	0.60	0.74	0.26
Angry-hostility	0.71	0.24	0.62	0.69	0.21
Depression	0.72	0.25	0.64	0.83	0.38
Self-consciousness	0.58	0.15	0.55	0.54	0.13
Impulsiveness	0.64	0.19	0.52	0.61	0.17
Vulnerability	0.77	0.30	0.62	0.79	0.32

*M_IC_, mean inter-item correlation*.

The M_IC_ of neuroticism subscale was 0.18 in the undergraduate sample and 0.21 in the clinical sample. In the undergraduate sample, the M_IC_ of the six facets ranged from 0.15 (self-consciousness) to 0.30 (vulnerability), while the clinical sample, the M_IC_ of six facets ranged from 0.13 (self-consciousness) to 0.38 (depression), Table 4.

In the undergraduate sample, the test-retest coefficient for the neuroticism subscale was 0.71, and the test-retest coefficients for the six facets ranged from 0.52 (impulsiveness) to 0.64 (depression), see Table 4. Test-retest reliability was not evaluated in the clinical sample.

### Intercorrelation among the total neuroticism subscale and its six facets

The correlation coefficients among the total score of neuroticism subscale and its six facets ranged from 0.44 to 0.85 in the undergraduate sample, and from 0.47 to 0.87 in the clinical sample (Table 5). All correlation coefficients were positive and statistically significant (*p* < 0.001).

**Table 5:** Intercorrelations among total neuroticism subscale and its six facets.

	**Undergraduate sample**						**Clinical sample**					
	**V**	**I**	**S**	**D**	**A^1^**	**A^2^**	**V**	**I**	**S**	**D**	**A^1^**	**A^2^**
I	0.56^**^						0.59^**^					
S	0.56^**^	0.37^**^					0.58^**^	0.47^**^				
D	0.67^**^	0.49^**^	0.60^**^				0.68^**^	0.54^**^	0.60^**^			
A^1^	0.55^**^	0.52^**^	0.44^**^	0.54^**^			0.59^**^	0.54^**^	0.54^**^	0.64^**^		
A^2^	0.68^**^	0.48^**^	0.56^**^	0.68^**^	0.56^**^		0.70^**^	0.54^**^	0.61^**^	0.77^**^	0.62^**^	
N	0.85^**^	0.72^**^	0.74^**^	0.84^**^	0.76^**^	0.83^**^	0.85^**^	0.75^**^	0.77^**^	0.87^**^	0.81^**^	0.87^**^

*V, Vulnerability; I, Impulsiveness; S, Self-consciousness; D, Depression; A^1^, Angry-Hostility; A^2^, Anxiety; N, Neuroticism*.

** *Correlation is significant at the 0.01 level (2-tailed)*.

### Gender differences and group differences in neuroticism

In the student subjects, females scored significantly higher than males on the total score of neuroticism subscale and scores of six facets (Total score: *t* = 17.031, *p* < 0.001, Cohen's *d* = 0.46; Angry-Hostility score: *t* = 11.775, *p* < 0.001, Cohen's *d* = 0.32; Anxiety score: *t* = 13.868, *p* < 0.001, Cohen's *d* = 0.37; Depression score: *t* = 11.197, *p* < 0.001, Cohen's *d* = 0.30; Self-Consciousness score: *t* = 9.688, *p* < 0.001, Cohen's *d* = 0.26; Impulsiveness score: *t* = 12.547, *p* < 0.001, Cohen's *d* = 0.34; and Vulnerability score: *t* = 21.358, *p* < 0.001, Cohen's *d* = 0.58). There were no significant differences in scores of neuroticism subscale and its six facets between males and females in the clinical sample (Table 6).Compared with the undergraduate group, the clinical group got significantly higher scores on total neuroticism (*t* = 35.70, *p* < 0.001, Cohen's *d* = 1.39) and all six facets (*t*: 19.06 ~ 33.96, all *p* < 0.001, Cohen's *d*: 0.80~1.31).

**Table 6:** Scores on total neuroticism subscale and each of the six facets separated by gender (Means ± SD, ranges).

**Scale**	**Undergraduate sample**				**Clinical sample**			
	**Total (*N* = 5,494)**	**Female (*N* = 2,616)**	**Male (*N* = 2,878)**	**Cohen's d**	**Total (*N* = 551)**	**Female (*N* = 270)**	**Male (*N* = 281)**	**Cohen's d**
N	72.12 ± 20.99^a^	77.05 ± 21.68^b^	67.64 ± 19.28	0.46	106.84 ± 28.36	106.02 ± 27.34	107.70 ± 29.40	–
	(11~155)	(11~155)	(12~146)		(19~175)	(19~175)	(30~174)	
A^1^	10.49 ± 4.60^a^	11.25 ± 4.88^b^	9.80 ± 4.22	0.32	16.66 ± 5.57	17.05 ± 5.71	16.29 ± 5.42	–
	(0~28)	(0~28)	(0~26)		(0~32)	(3~32)	(2~30)	
A^2^	12.57 ± 4.41^a^	13.42 ± 4.56^b^	11.80 ± 4.41	0.37	19.13 ± 5.85	19.42 ± 5.93	18.85 ± 5.77	–
	(0~30)	(0~30)	(0~26)		(1~32)	(1~32)	(1~32)	
D	11.67 ± 4.71^a^	12.41 ± 4.84^b^	11.00 ± 4.50	0.30	18.24 ± 6.68	18.08 ± 7.16	18.39 ± 6.18	–
	(0~32)	(0~32)	(0~27)		(0~32)	(0~32)	(0~32)	
S	13.14 ± 4.12^a^	13.70 ± 4.28^b^	12.63 ± 3.91	0.26	17.92 ± 5.27	18.07 ± 5.30	17.78 ± 5.24	–
	(0~29)	(0~29)	(0~28)		(3~47)	(5~47)	(7~32)	
I	13.20 ± 4.33^a^	13.96 ± 4.46^b^	12.51 ± 4.09	0.34	16.95 ± 5.06	16.75 ± 5.35	17.13 ± 4.77	–
	(0~28)	(1~28)	(0~27)		(0~29)	(2~29)	(8~29)	
V	11.05 ± 4.36^a^	12.32 ± 4.39^b^	9.90 ± 3.99	0.58	17.94 ± 6.06	18.32 ± 6.04	17.57 ± 6.07	–
	(0~31)	(0~31)	(0~23)		(0~32)	(0~32)	(0~32)	

*N, neuroticism; A^1^, Angry—Hostility; A^2^, Anxiety; D, Depression; S, Self-Consciousness; I, Impulsiveness; V, Vulnerability*.

a Clinical sample scored significantly higher than undergraduate sample, p < 0.05;

b *Women scored significantly higher than men, p < 0.05*.

### Predictive validity of demographic variables and the six facets of neuroticism subscale for depression and anxiety

Results of multiple regression analyses with the CES-D total score as dependent variable were presented in Table 7. For the undergraduate sample, demographic variables (gender and age), anxiety facet, angry-hostility facet, depression facet, self-consciousness facet and vulnerability facet significantly predicted the CES-D total score (*p* < 0.05), except impulsiveness facet (*p* > 0.05). For the clinical sample, anxiety facet, depression facet and vulnerability facet significantly predicted the CES-D total score (*p* < 0.05), but none of other three facets of the neuroticism subscale significantly predicted the CES-D total score (*p* > 0.05).

**Table 7:** Multiple regression analysis with the CES-D total score as the dependent variable.

	**Undergraduate sample (*N* = 5,494)**				**Clinical sample(*N* = 551)**			
	**ß**	**95%CI**	***t***	***p***	**ß**	**95%CI**	***t***	***p***
Gender	−0.03	−0.68~–0.06	−2.30	0.02	0.02	−1.17~1.90	0.47	0.64
Age	−0.02	−0.32~–0.02	−2.25	0.02	−0.02	−0.05~0.03	−0.64	0.52
Anxiety	0.18	0.25~0.36	11.52	< 0.01	0.23	0.19~0.65	3.62	<0.01
Angry-hostility	0.09	0.10~0.19	6.53	<0.01	0.08	−0.04~0.36	1.55	0.12
Depression	0.31	0.43~0.53	18.98	<0.01	0.20	0.11~0.51	3.10	<0.01
Self-consciousness	0.03	0.01~0.10	2.34	0.02	−0.03	−0.27~0.14	−0.65	0.52
Impulsiveness	0.02	−0.01~0.08	1.50	0.13	−0.04	−0.29~0.11	−0.87	0.39
Vulnerability	0.13	0.17~0.28	7.97	<0.01	0.20	0.16~0.55	3.52	<0.01
Constant		61.04~649.47	2.37	0.02		−25.12~129.86	1.32	0.19
	*F* = 483.42; *p* < 0.01; *R*^2^ = 0.41				*F* = 31.97; *p* < 0.01; *R*^2^ = 0.33			

ß*, standardized ß; 95%CI, 95% confidence interval for ß*.

As Table 8 showed, for the undergraduate sample, demographic variables (gender and age), anxiety facet, angry-hostility facet, depression facet, impulsiveness facet and vulnerability facet all significantly predicted the SAS total score (*p* < 0.05), except self-consciousness facet (*p* > 0.05). For the clinical sample, anxiety facet, angry-hostility facet and vulnerability facet significantly predicted the SAS total score (*p* < 0.05), while none of other three facets of the neuroticism subscale significantly predicted the SAS total score (*p* > 0.05).

**Table 8:** Multiple regression analysis with the SAS total score as the dependent variable.

	**Undergraduate sample (*N* = 5,494)**				**Clinical sample(*N* = 551)**			
	**ß**	**95%CI**	***t***	***p***	**ß**	**95%CI**	***t***	***p***
Gender	−0.03	−0.70~–0.02	−2.10	0.04	−0.01	−1.75~1.41	−0.22	0.83
Age	−0.04	−0.43~–0.11	−3.25	<0.01	−0.07	−0.08~0.00	−1.86	0.06
Anxiety	0.22	0.30~0.42	12.48	<0.01	0.23	0.17~0.64	3.41	<0.01
Angry-hostility	0.16	0.20~0.29	10.43	<0.01	0.20	0.17~0.58	3.60	<0.01
Depression	0.15	0.17~0.28	8.21	<0.01	0.00	−0.20~0.20	−0.01	1.00
Self-consciousness	0.01	−0.04~0.07	0.54	0.59	0.01	−0.20~0.22	0.11	0.92
Impulsiveness	0.11	0.13~0.23	7.29	<0.01	0.06	−0.09~0.32	1.08	0.28
Vulnerability	0.05	0.03~0.15	2.93	<0.01	0.16	0.08~0.49	2.76	<0.01
Constant		235.77~878.04	3.40	<0.01		17.00~176.16	2.39	0.02
	*F* = 300.84; *p* < 0.01; *R*^2^ = 0.30				*F* = 26.78; *p* < 0.01; *R*^2^ = 0.29			

ß*, standardized ß; 95%CI, 95% confidence interval for ß*.

## Discussion

The neuroticism subscale of the NEO-PI is widely used around the world and is considered a valid and reliable measurement for the personality trait of neuroticism (Costa and McCrae, 1992a; Nunnally, 1994). The present study evaluated the reliability and validity of the neuroticism subscale in two Chinese samples (an undergraduate sample and a clinical sample), and examined its measurement invariance across gender in the undergraduate sample. Three hypothetical models were tested: single-factor model, six-facet model and bi-factor model (six-facet model with one general factor). The model indices of the six-facet model and the bi-factor model for both undergraduate sample and clinical sample all met the fit standards, supporting the neuroticism subscale's construct validity. Since the six-facet model fitted the data best, the six-facet model was chosen in the following measurement invariance analysis. In the undergraduate sample, the model indices for both the males and females indicated a good fit of the neuroticism subscale's theoretical structure. In addition, the Chinese version of the neuroticism subscale also demonstrated acceptable reliability and factorial validity in this study.

The Cronbach's α coefficients for the total neuroticism subscale, in both the undergraduate sample and the clinical sample, reached accepted standards (α > 0.90). Among the six facets, 5 of them were above 0.60 in the two samples, while the self-consciousness facet was with a value of < 0.60. These results were consistent with those of the original English version, which reported the internal reliability of all facets ranged from 0.58 to 0.81 (Costa and McCrae, 1992a). Hence, the α values found in this study were deemed acceptable. Although the α of self-consciousness facets in this study were similar to those of the original version (Costa and McCrae, 1992a), the internal consistency of self-consciousness facet should be reexamined in the future, and researchers should be cautious in interpreting this facet. In both the undergraduate sample and the clinical sample, all the mean inter-item coefficients were above the lowest accepted level (Briggs and Venue, 1986), which also supports good internal consistency of the neuroticism subscale of the NEO-PI.

The test-retest reliability coefficient for the total neuroticism subscale was high and supports the notion of neuroticism as a stable personality trait. These findings were in agreement with the original version of NEO-PI (Anastasi and Urbina, 1997). The 2-month stability coefficients of the six facets ranged from 0.52 to 0.64 in this study, while a previous study found that the 6-month test-retest reliability of the neuroticism subscale of NEO-PI ranged from 0.66 to 0.92 (Costa and McCrae, 1988). Our results were a little lower than those of the previous study, which might be due to natural fluctuations in the state of the six facets of neuroticism. Further studies are needed to examine the stability of neuroticism subscale in both general population and clinical population, with larger samples and longer interval.

The correlations among the six facets were all significantly positive in both two groups (all *p* < 0.01), which indicated that the six facets not only possessed relative independence, but also related to each other. The six facets of the neuroticism subscale measure different aspects of neuroticism, while all reflect the neuroticism trait. Anyway, these results provided further support for the validity of the neuroticism subscale.

The scores on total neuroticism subscale and all six facets were significantly higher in the clinical sample than the undergraduate sample. These results were consistent with previous findings (Kotov et al., 2010; Ormel et al., 2013), for example, Kotov et al. found that individuals diagnosed with mental disorders also scored high in neuroticism (Kotov et al., 2010). Previous studies also supported that neuroticism is correlated with a wide variety of mental disorders, and individuals with high neuroticism tend to have more mental disorders (Kotov et al., 2010; Ormel et al., 2013). It has previously been stated that neuroticism is one of the most important risk factors in behavioral public health (Lahey, 2009), and that the economic costs of high neuroticism are estimated to exceed those of all common mental disorders combined (Cuijpers et al., 2010). Additionally, it has been shown that neuroticism could predict the onset of common mental disorders even after controlling for most psychiatric confounding variables (Ormel et al., 2013). All these findings suggest the importance of neuroticism in screening individuals with high-risk mental disorders, and in the implementation of early prevention programs to individuals with high-level neuroticism.

The current study found that most of facets could significantly predict depression and anxiety symptoms both in the undergraduate sample and clinical sample, especially the anxiety facet. Previous research has shown a strong link between personality and psychiatric illness, especially in the relationship between neuroticism and depressive symptoms (Luciano, 2015). Individual differences in personality traits, particularly in neuroticism, are known risk factors in the onset and development of depression (Saklofske and Janzen, 1995; Kendler et al., 2004). Research in England indicated that neuroticism predisposes individuals to depression via the relationship between ruminative thinking and low mood (Thorsten and Tobias, 2010). Additionally, certain personality traits may play an important role in how individuals respond to life-events. Individuals with high level of neuroticism are particularly vulnerable to the effect of life events on anxiety (Veen et al., 2016). A study, investigating the relationship between neuroticism and symptoms of anxiety and depression in three patient groups (generalized anxiety disorder; major depressive disorder; mixed anxiety-depressive disorder), revealed that neuroticism might increase the risk of anxious and depressive symptoms, as evidenced by increased worrying or brooding (Merino et al., 2016). While in this study, several facets (anxiety, depression and vulnerability) of the neuroticism subscale could significantly predict the depression level, and three facets (anxiety, angry-hostility and vulnerability) could significantly predict the anxiety level. Therefore, our results, in conjunction with the prior researches discussed above, supported that neuroticism has a great impact on symptoms of depression and anxiety both in general population and in those diagnosed with mental disorders.

It should be stressed that the validity of comparing groups is dependent on measurement invariance. The examination of measurement invariance is a necessary step prior to performing any comparisons across groups (Meredith, 1993; Byrne, 2008). The measurement invariance across gender in the current study supported weak equivalence (equal factor loadings) and strong equivalence (equal factor loadings /intercepts), which is sufficient for meaningful comparisons between groups. No strict measurement invariance was get in our results, while as Baumgartner suggested, strict measurement invariance is not required for substantive analyses (Baumgartner and Steenkamp, 1998). In general, the results of measurement invariance across gender in this study supported the validity of the significant gender differences in neuroticism scores, which was consistent with previous findings (Hirsh, 2011). Our results showed that women scored significantly higher than men on both total neuroticism and all six facets in the undergraduate sample, while in the clinical sample, there were no significant gender differences in total score of neuroticism or scores of its six facets. In the current study, the high level of neuroticism and relatively small size of the clinical sample may contribute to the lack of gender differences.

## Conclusion

The current study provides further evidence that the neuroticism subscale of the NEO-PI is a reliable and valid measurement tool for assessing neuroticism in Chinese population. Additionally, its measurement invariance across gender supported that the gender differences in neuroticism found by the Chinese version of the neuroticism subscale of the NEO-PI were reliable and valid.

Our research was restricted by specific Chinese samples, and the generalizability of results in this study to other countries remains to be determined. Studies with longitudinal research design and with larger clinical sample sizes are warranted.

## Author contributions

JY and SY: conceived and designed the study; MZ, XL, YiL, YuL and XZ: collected the data; CX, MZ, XL, and YiL: analyzed the data; CX and JY: wrote the paper.

### Conflict of interest statement

The authors declare that the research was conducted in the absence of any commercial or financial relationships that could be construed as a potential conflict of interest.
