# Supplementary material for: Psychometric Properties of the Chinese Version of the Neuroticism Subscale of the NEO-PI
Source: Front Psychol. 2018 Aug 17;9:1454. doi: 10.3389/fpsyg.2018.01454 (PMC6108233; doi:10.3389/fpsyg.2018.01454)
Supplement: Supplementary file 1 [file Presentation_1.pdf]

Supplementary Material

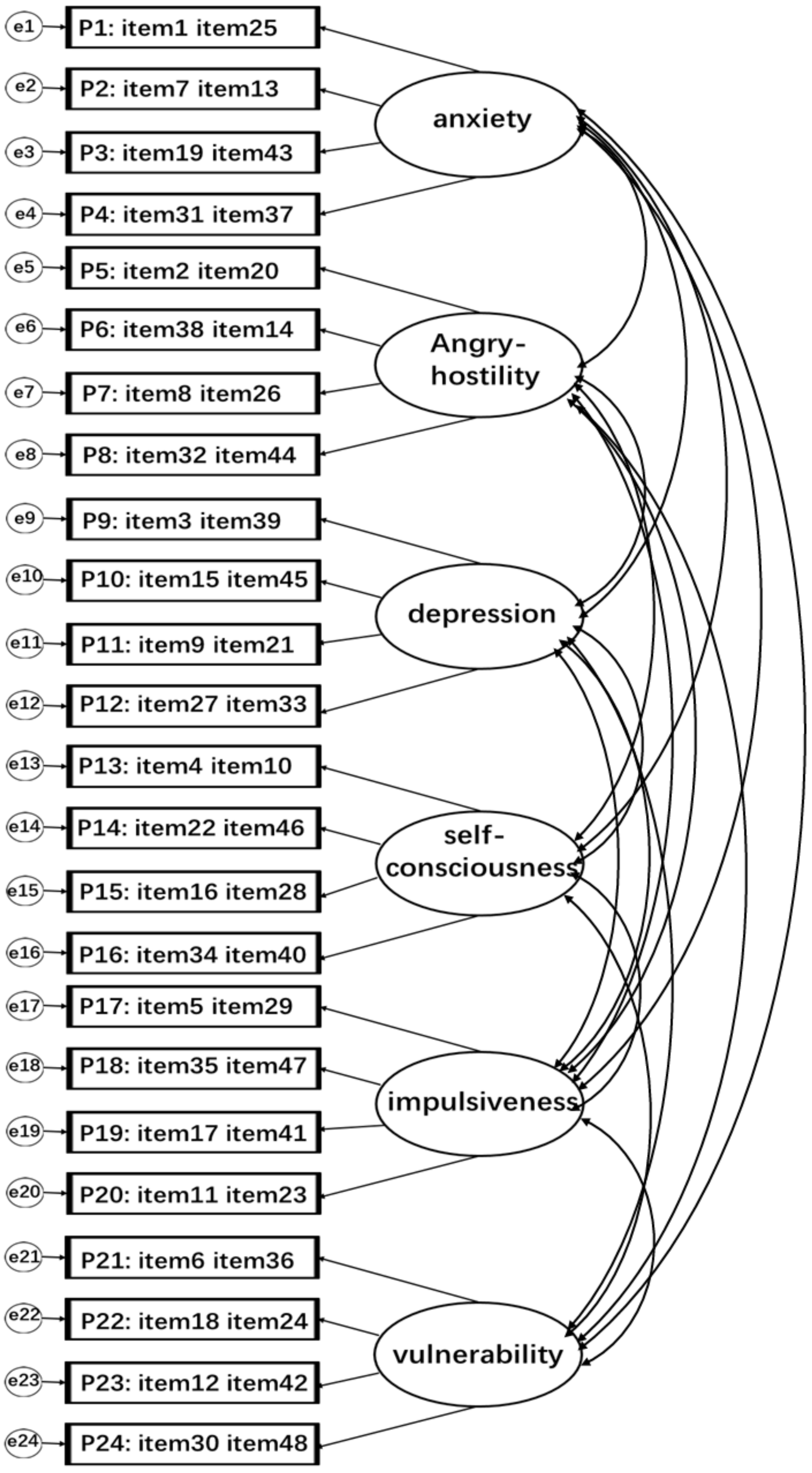

S-Figure 1. Structural model of six facets

Note: Each facet consists of 4 parcels; “P1: item1 item25” means parcel 1 including item 1 and item 25.
